# The dynamics of auditory working memory impairment in logopenic variant primary progressive aphasia and typical Alzheimer’s disease

**DOI:** 10.1002/alz.091482

**Published:** 2025-01-03

**Authors:** Benjamin Aaron Levett, Catalina Novoa, Lucy Core, Jessica Jiang, Jason D Warren, Chris JD Hardy

**Affiliations:** ^1^ Dementia Research Centre, UCL Queen Square Institute of Neurology, University College London, London, London United Kingdom; ^2^ Dementia Research Centre, UCL Queen Square Institute of Neurology, University College London, London United Kingdom; ^3^ Dementia Research Centre, Department of Neurodegenerative Disease, UCL Queen Square Institute of Neurology, University College London, London United Kingdom

## Abstract

**Background:**

Impaired auditory verbal working memory is a diagnostic hallmark and integral driver of the clinical phenotype in logopenic variant primary progressive aphasia (lvPPA). However, the physiology of the working memory buffer in this syndrome is poorly characterised. Here we addressed the temporal dynamics of auditory verbal working memory in patients with lvPPA and typical Alzheimer’s disease (tAD).

**Method:**

In a cohort of 8 patients with lvPPA, 17 patients with tAD and 18 healthy age‐matched controls, we assessed how temporal manipulations of a standard auditory verbal working memory tasks (forward digit span and phrasal repetition) affected performance. We varied tempo of delivery and inter‐trial gap and performed a detailed analysis of error types. Participants also underwent pure tone audiometry to assess peripheral hearing function and a comprehensive general neuropsychological assessment.

**Result:**

Compared with healthy controls, patients with lvPPA and tAD showed increased sensitivity to temporal manipulation of auditory verbal working memory tasks and error profiles suggesting a dynamic physiological lesion of the working memory buffer. These effects were particularly marked in the lvPPA group.

**Conclusion:**

Our findings open a novel physiological window on working memory dynamics in Alzheimer’s disease syndromes. Further work is warranted to assess how this dynamic deficit impacts communication in patients’ daily lives, how it can best inform management interventions and its potential as a novel, rapid read‐out of neural function in the era of disease‐modifying therapies.

